# Comparing dialysis centre mortality outcomes across Australia and New Zealand: identifying unusually performing centres 2008–2013

**DOI:** 10.1186/s12913-018-3832-0

**Published:** 2018-12-29

**Authors:** Jessica Kasza, Kevan R. Polkinghorne, Rory Wolfe, Stephen P. McDonald, Mark R. Marshall

**Affiliations:** 1 Department of Epidemiology and Preventive Medicine, Monash University, Melbourne, Victoria 3004 Australia; 2 Departments of Nephrology and Medicine, Monash Medical Centre, Monash University, Melbourne, Victoria 3168 Australia; 3 ANZDATA Registry, SA Health and Medical Research Institute, Adelaide, South Australia 5000 Australia; 4 Discipline of Medicine, University of Adelaide, Adelaide, South Australia 5005 Australia; 5 Department of Medicine, Faculty of Medical and Health Sciences, University of Auckland, Private Bag 92019, Auckland, 1142 New Zealand; 6 Baxter Healthcare (Asia) Pte Ltd, 150 Beach Road. #30-01/08 Gateway West, Singapore, 189720 Singapore; 7 Department of Renal Medicine, Counties Manukau Health, Private Bag 93311, Auckland, 1640 New Zealand

**Keywords:** Center performance, Mortality, Transplantation, Standardized mortality ratio

## Abstract

**Background:**

Comparing the mortality profiles of dialysis centres is important to ensure that high standards of care are maintained. We compare the performance of dialysis centres in Australia and New Zealand in their treatment of haemodialysis patients, accounting for the competing risks of kidney transplantation and transfer to peritoneal dialysis.

**Methods:**

Observational cohort study. We included data from all adult patients (5574 patients) commencing haemodialysis at home or in a facility between 2008 and 2010 across 62 dialysis centres, from the Australia and New Zealand Dialysis and Transplant Registry. Standardised mortality ratios were calculated by estimating mortality probabilities from a pooled random effects logistic regression model, accounting for the competing risk of transplantation using an inverse probability weighting approach. Models were adjusted for patient comorbidities, sex, height, weight, late referral to a nephrologist, age, race, primary kidney disease, smoking status, and serum creatinine (μmol/l).

**Results:**

Two dialysis centres were found to have relatively higher levels of risk-adjusted mortality lying outside the prediction intervals for “usual” performance. Risk adjusted mortality rates were not associated with centres’ compliance with guidelines for vascular access and biochemical and haematological targets.

**Conclusions:**

We demonstrate that standardised mortality ratios are useful to identify facilities that have statistically outlying mortality risk. Our criterion for determining whether a centre has better or worse performance than expected is statistical, and thus analyses such as ours can serve only as a screening tool, and are only one aspect of assessment of “quality” of performance.

**Electronic supplementary material:**

The online version of this article (10.1186/s12913-018-3832-0) contains supplementary material, which is available to authorized users.

## Background

Quality assurance requires the ability to measure clinical outcomes against a standard, and the ability to alter processes to measurably affect outcomes. In order to ensure a high quality of care for dialysis patients, it is necessary to identify dialysis centres in which “performance” may be unusually poor or excellent. Such identification is of interest to stakeholders at all levels, from patients through to regulatory authorities, and is a fundamental step that defines the need and form of action to be taken.

In this article, we illustrate how performance may be measured and compared across dialysis centres, using the outcome of mortality of haemodialysis patients. We emphasise the difficulty in determining whether unusual levels of mortality, where “unusual” is defined using statistical criteria, is reflective of truly anomalous performance. Mortality is only one element of overall “quality” of dialysis, and is the outcome of complex and multifactorial pathways. We do not here seek to compare the overall “quality” of dialysis centres: such an exercise requires consideration of multiple aspects of processes of care and patient outcomes [1].

Currently, the Australia and New Zealand Dialysis and Transplant (ANZDATA) Registry, [2], provides annual performance reports to individual contributing hospitals: examples of these reports are available online (http://www.anzdata.org.au/v1/dialysis_hospitalreport.html), and include risk-adjusted mortality estimates. All dialysis centres within Australia and New Zealand contribute to the ANZDATA Registry, and we explored the risk-adjusted mortality of haemodialysis patients treated within Australian and New Zealand dialysis centres, utilising more sophisticated methods of handling competing risks and estimation of standard errors than currently implemented by ANZDATA. Given the differences in treating patients on peritoneal dialysis and haemodialysis, we restrict our focus to haemodialysis patients.

## Methods

Data for all patients aged over 18 years who commenced haemodialysis between January 1, 2008 and December 31, 2010 was extracted from the ANZDATA Registry: at the time of data extraction, this was the most recent data to allow for 3 years of follow-up. Patients who were undergoing home haemodialysis were included in the analysis, as were all patients who switched between facility-based haemodialysis and home-based haemodialysis. Patients were followed until death, loss to follow up, kidney transplantation, regain of kidney function, or start of peritoneal dialysis (PD; centre or home-based). Time to mortality was the outcome of interest with censoring at the time of one of these other events or at 3 years after dialysis start. Patients were excluded from the analysis if they had fewer than 90 days of dialysis, or if they commenced PD within 30 days of dialysis start. Patients who underwent kidney transplantation prior to receiving any dialysis were not included in our analysis. Patient outcomes were assigned to the last centre that had provided dialysis care for at least 30 days at the time of death, loss to follow up, kidney transplant, regain of kidney function, transfer to peritoneal dialysis, or at three years after dialysis start [3, 4].

We excluded centres that treated fewer than 10 haemodialysis patients to ensure stability of estimates, and centres that ceased or commenced operation between January 1, 2008 and December 31, 2013. The exception to this was when centres amalgamated: in this situation, all patients at the amalgamating centres were assigned to the amalgamated centre. Patients who had their care transferred to a centre that commenced operation between January 1, 2008 and December 31, 2013 had their survival time censored at 30 days after the switch, to allow for any possible carry-over effects of treatment attributable to their previous centre.

At dialysis start, ANZDATA records: comorbidities (diabetes mellitus, coronary artery, peripheral vascular, cerebrovascular and chronic lung diseases), sex, height, weight, late referral to a nephrologist (first seen by a nephrologist < 3 months before dialysis start), age, race (categories consolidated as Caucasian, Asian, Maori/Pacific Islander/Aboriginal or Torres Strait Islander, Indian/Arabic, Other), primary kidney disease (categories consolidated as diabetes mellitus, glomerulonephritis, hypertensive nephropathy, polycystic kidney disease, other), smoking status (current, former, never), and serum creatinine (μmol/l). Diabetes mellitus was coded as type I, II, or none, and all other comorbidities were recorded as yes, suspected, or no (yes and suspected categories were combined for analysis). Body mass index (BMI, in kg/m^2^) was calculated for each patient from height and weight recorded at dialysis start.

## Statistical methods

### Prediction model for mortality and risk adjustment

In order to calculate mortality performance indicators for each dialysis centre (the standardised mortality ratios described below), predictions of mortality probabilities for each patient are required. To obtain these probabilities, pooled logistic regression models were fit after discretising patient survival time into 30 day periods. Such models allow the calculation of weights accounting for informative censoring in the manner described below, and approximate Cox proportional hazards models [5]. These models included random effects for centres, as has been recommended for healthcare provider comparison [6]. Patient characteristics recorded at the time of dialysis start listed above, year of dialysis start, and country (Australia or New Zealand) were included in the model. Characteristics of centres and centre-level summary statistics of patients under the control of the centre may be the cause of any observed unusually poor or good performance, and thus were not included in the model [6].

### Accounting for informative censoring: Kidney transplant or switch to peritoneal dialysis

Patients who switch to PD or undergo a kidney transplant are likely to be quite different from those patients who remain on haemodialysis, and patterns of treatment switching and kidney transplantation are likely to differ between dialysis centres: thus it is necessary to account for this informative censoring to obtain unbiased standardised mortality ratios (SMRs). We used a weighting approach to account for this issue: fitting pooled logistic regression models the probability of a kidney transplant, and the probability of a switch to peritoneal dialysis [7]. These models included the same set of covariates as included in the model for mortality. When fitting the model for mortality, each patient’s observation at each time point was then weighted by his/her inverse probability of remaining untransplanted and on haemodialysis [8]. In this way a “pseudo-population” in which no patients received a kidney transplantation or switched to PD is generated [9]. This approach assumes that all confounders of the informative-censoring and mortality relationship are measured and included in the pooled logistic regression models.

### Performance indicator

As with other studies comparing performance of dialysis [10, 11] and transplantation centres [12], we calculate and compare standardised mortality ratios (SMRs) for each centre, and apply statistical criteria to identify those that have poor performance. Generally, the SMR of a centre is the ratio of the observed number of deaths within that centre to the number of deaths expected, defined as the number of deaths that would have been observed if the patients treated at that centre had been assigned at random to other centres [13]. A formula is provided in Additional Item 1 in Additional file 1. The logarithm of the SMR was taken to obtain confidence intervals with good coverage properties, and standard errors of log-SMRs were obtained by bootstrapping [14, 15]. Funnel plots were used to display results [16], with log-SMRs plotted against the “effective sample size”, a measure of the variability of each log-SMR relative to the overall variability of all log-SMRs [17]. Due to the multiplicity of hypotheses being considered (for each centre that their log-SMR is equal to 0), we controlled the family-wise error rate and the false discovery rate [18]. Prediction limits corresponding to both of these methods of multiplicity control, and those corresponding to the classical 0.05 level, are drawn on the funnel plots.

For comparison, we also calculated log-SMRs using an un-weighted model. A sensitivity analysis was conducted where all patients commencing peritoneal dialysis within 90 days of dialysis start were excluded from the calculation of log-SMRs. Since there is some controversy surrounding whether or not race should be adjusted for in the comparison of provider performance [19], we also consider log-SMRs calculated when race is excluded from the models for weights and the model for mortality.

### Compliance of centres with guidelines

We sought to determine whether the observed performance of centres as measured by the log-SMR is associated with compliance with Kidney Health Australia – Caring for Australasians with Renal Impairment (KHA-CARI) Guidelines for vascular access, biochemical and haematological targets [20, 21]. For each centre, we calculated: the proportion of haemodialysis patients with an arteriovenous fistula; the proportion of calcium measurements between 2.1 and 2.4 mmol/L; the proportion of phosphate measurements between 0.8 and 1.6 mmol/L; the proportion of serum ferritin measurements between 200 and 500 μg/L; and the proportion of haemoglobin measurements between 100 and 115 g/L.

## Results

Eight thousand seven hundred fifty-two patients commenced dialysis in Australia and New Zealand between January 1, 2008 and December 31 2010. Seven Hundred Fifty-One patients had fewer than 90 days of dialysis, 70 commenced dialysis at the seven centres that were not in operation for the entire period of observation, 2288 patients commenced PD within 30 days of dialysis start, 38 commenced dialysis in the six centres that had fewer than 10 patients, and 31 patients had missing data for one or more covariates. The remaining 5574 patients were under the care of the 62 centres that treated more than 10 patients and were in operation for the entire observation period: we calculate SMRs for these 62 centres. Of these 5574 patients, 1295 died, 545 received a kidney transplant, and 843 commenced peritoneal dialysis after day 30 of haemodialysis, within 3 years of dialysis start.

Figure 1 indicates that the mortality and transplantation/regain of kidney function rates unadjusted for patient characteristics vary markedly across centres. However, given that patient characteristics also vary across centres (Table 1 and Additional file 1: Figures S1–9 ), the raw mortality ratios will not be useful in the comparison of mortality rates across centres.

**Fig. 1:**
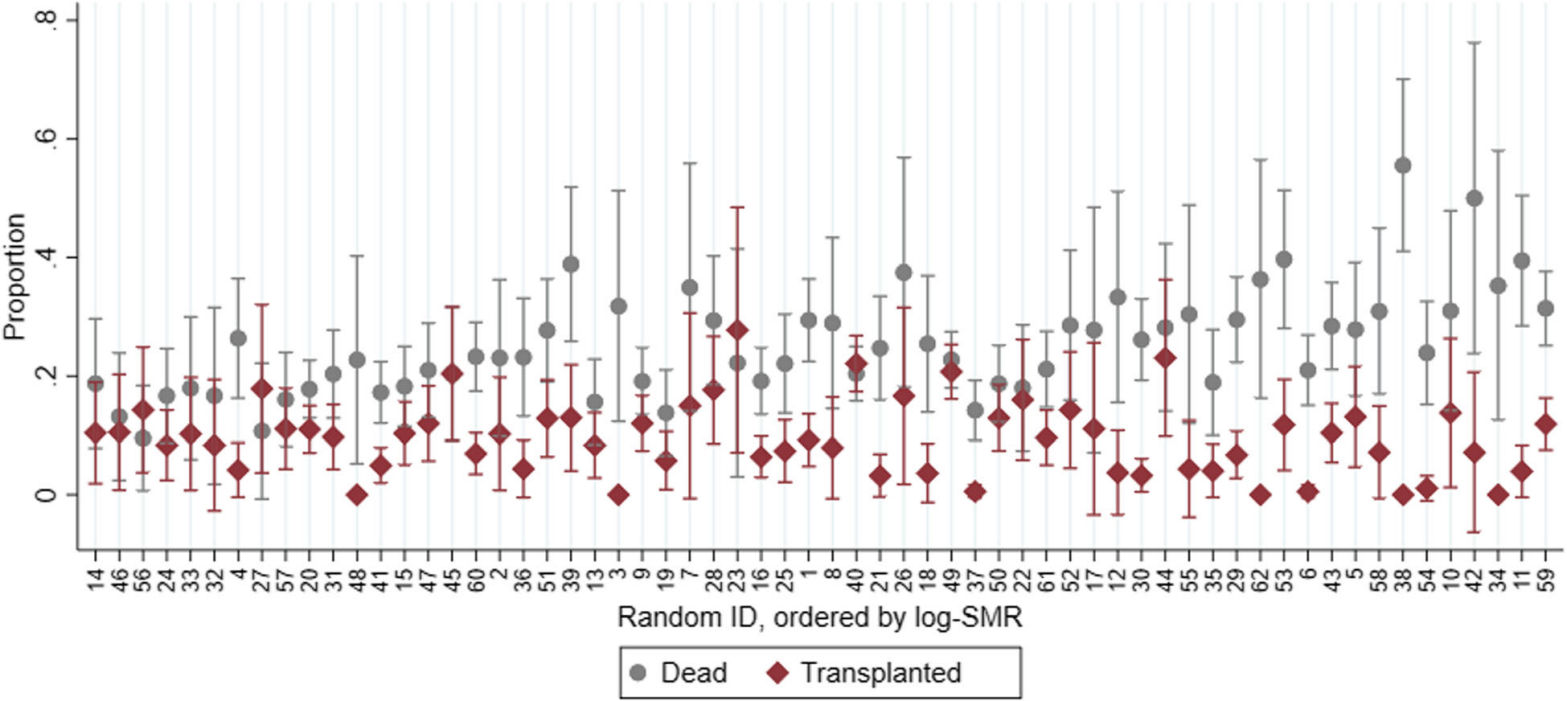
Proportion of patients within each dialysis centre dying or receiving kidney transplants/regaining kidney function, with 95% confidence intervals. Centres are ordered by the log-SMR (smallest to largest) and are labelled with random identifying numbers

**Table 1:** Summaries of centre-level means and percentages of patient characteristics. The percentage of patients in each category are presented unless otherwise indicated

Characteristic	Minimum	Lower quartile	Median	Upper quartile	Maximum
Mean age at Renal Replacement Therapy (years)	49.2	58.6	62.4	64.9	76.8
Mean BMI (kg/m^2^)	25.1	27.4	28.4	29.9	33.6
Mean serum creatinine at entry (μmol/L)	375.7	599.6	664.4	744.5	942.5
Male	40.9	58.0	61.2	66.3	74.4
Late referral to nephrologist	10.0	18.0	26.6	30.1	53.8
Lung disease	3.4	13.2	17.5	22.7	82.4
Peripheral vascular disease	4.8	18.8	28.3	35.4	82.4
Cerebrovascular disease	4.8	11.4	15.6	21.6	64.7
Coronary artery disease	18.5	38.0	44.5	55.0	88.2
Diabetic status					
No diabetes	6.5	45.1	54.5	61.9	76.9
Type I	0.0	1.1	2.2	3.5	11.1
Type 2	16.7	34.8	44.0	51.0	93.5
Year of dialysis start					
2008	11.1	28.9	34.5	40.2	59.1
2009	17.4	28.6	32.6	36.8	49.1
2010	15.0	28.1	31.9	36.4	65.2
Race					
Caucasoid	0.0	69.0	83.3	93.1	100.0
Asian	0.0	0.0	1.7	5.0	15.0
Maori/Pacific Is/Aboriginal	0.0	2.0	6.9	24.0	100.0
Indian/Arabic	0.0	0.0	2.1	4.0	8.7
Other	0.0	0.0	0.5	2.0	6.0
Smoking status					
Never	20.0	35.4	42.1	47.6	77.9
Former	19.1	38.9	45.6	51.7	62.5
Current	0.0	8.3	12.7	16.7	28.6
Primary renal disease					
Diabetes Mellitus	7.1	27.2	33.5	42.4	83.7
Glomerulonephritis	0.0	15.9	19.2	25.0	37.0
Hypertensive nephropathy	2.7	9.1	13.8	18.6	57.1
Other	5.5	16.2	22.5	29.2	55.6
Polycystic kidney disease	0.0	2.7	5.4	8.0	22.7

**Fig. 2:**
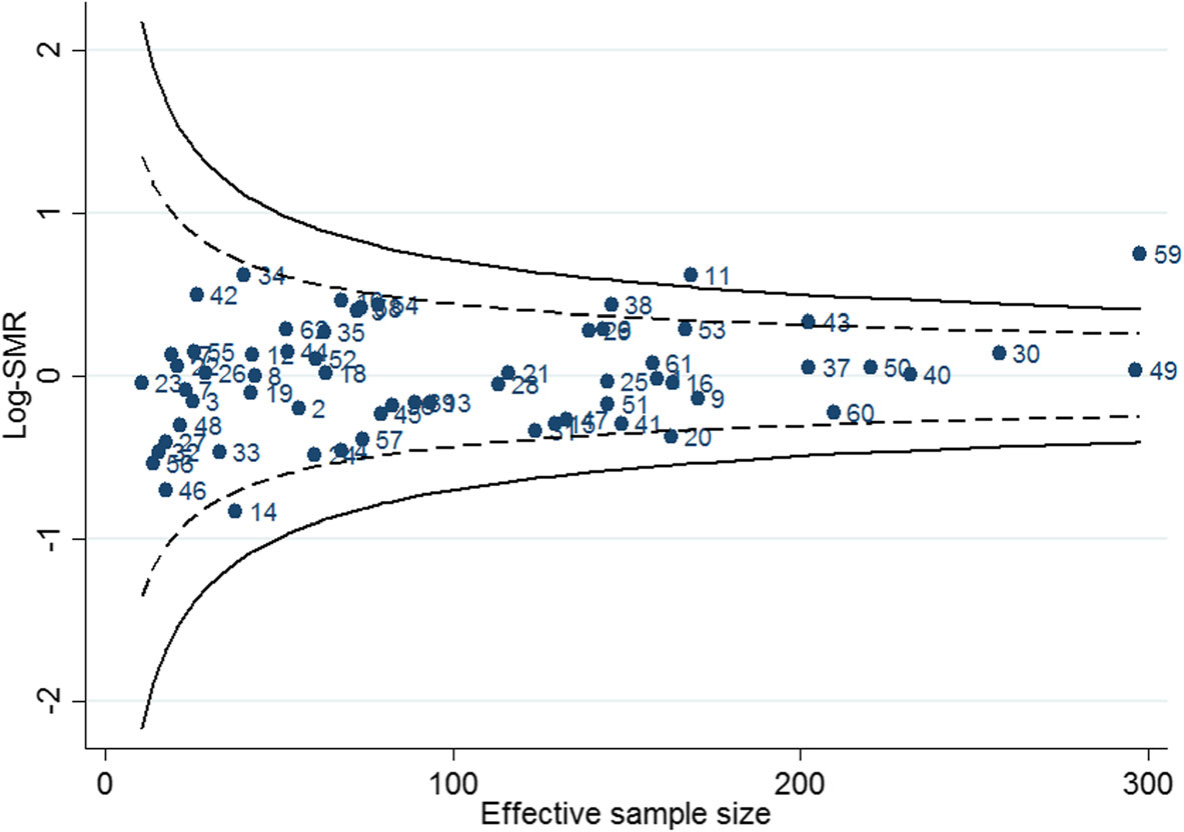
Funnel plot for log-SMRs obtained using the random effects pooled logistic model for mortality, accounting for censoring at the time of kidney transplant and switch to peritoneal dialysis using the weighting approach. The dashed lines are the classical 95% prediction limits when multiple comparisons are not adjusted for; the solid lines control the false discovery rate at 5%. Centres are labelled with random identifying numbers

Figure 2 displays the funnel plot for log-SMRs calculated when random effects were included for centres in the mortality prediction model and censoring due to transplantation and treatment switching to PD were dealt with using the weighting approach. When either the family-wise error rate or the false discovery rate was controlled at 5%, centres 11 and 59 were identified as having unusually large log-SMRs, i.e. mortality rates that were statistically higher than expected. Similar results were obtained when the informative censoring of patients at the time of a switch to PD or kidney transplantation was not accounted for using the weighting approach (Additional file 1: Figure S10). When patients who commenced peritoneal dialysis within 90 days of dialysis start (instead of excluding those patients who commenced within 30 days of dialysis start), the results are similar, although only centre 59 continued to display a significantly high log-SMR (Additional file 1: Figure S11). When race was excluded from the risk-adjustment models, the results were similar to those obtained for the primary analysis (Additional file 1: Figure S12).

**Fig. 3:**
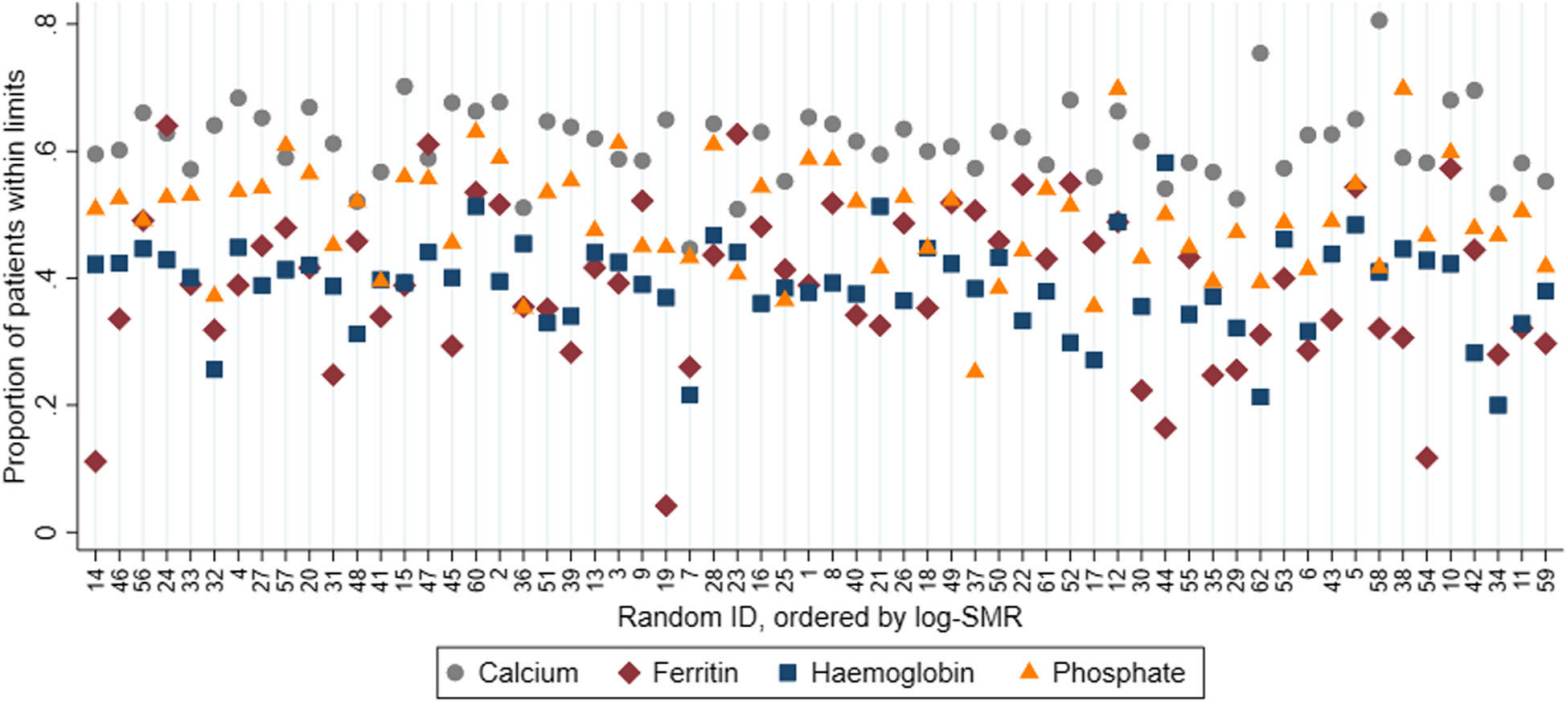
Proportion of measurements consistent with KHA-CARI guidelines for calcium, ferritin, haemoglobin and phosphate concentrations, within each dialysis centre. Centres are ordered by the log-SMR (smallest to largest) and labelled with random identifying numbers

Figure 3 displays the proportions of recorded laboratory measurements within each of the recommended limits for each centre. For each laboratory measurement, centre 59 appears to have a somewhat low proportion of patients within the recommended limits, particularly when compared only to Australian centres. However, the proportions of laboratory measurements within the recommended guidelines is not particularly unusual for either centre 59 nor 11.

**Fig. 4:**
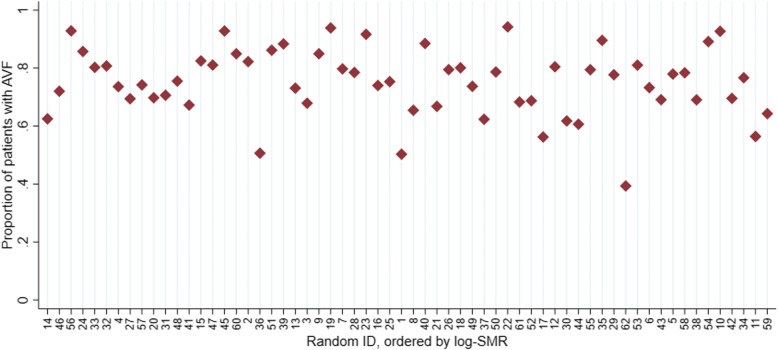
Proportion of patients with arteriovenous fistulas within each dialysis centre. Centres are ordered by the log-SMR (smallest to largest) and labelled with random identifying numbers

Figure 4 displays the proportions of patients with arteriovenous fistulas in each centre. Centre 11 appears to have a relatively low proportion of patients with arteriovenous fistulas than in other centres of similar size. Figures 3 and 4 indicate that just as for mortality, there is a large amount of variation between centres with respect to the proportion of measurements within recommended targets.

Further analysis, comparing log-SMRs to each of the proportions in Figs. 3 and 4 directly using scatter plots (Additional file 1: Figures S13-14), indicate that there does not appear to be any evidence of an association between each of these proportions and risk-adjusted mortality as measured by the log-SMR.

## Discussion

Our analysis identified two dialysis centres as having unusual performance, when characterising performance using the log-SMR. These centres do not appear to have markedly different mixes of patient ages, serum creatinine levels, BMIs, proportions of patients with each considered comorbidity, race, or primary kidney disease, or markedly different proportions of patients with late referral when compared to the other centres (Additional file 1: Figures S1–8), nor do they have any unusual patterns of missing data or proportions of measurements within recommended guidelines. Centre 59 does have the largest effective sample size of all centres considered in this study, and is one of the largest centres in terms of the number of patients. Although we cannot definitively determine whether this centre has truly poor performance, it is possible that the apparent poor performance of this centre may be in part due centre characteristics such as staffing levels. Further, that other similarly large SMRs from other centres did not reach statistical significance could be explained by a lack of power due to smaller effective sample sizes.

That these centres are identified as having unusual performance in mortality outcomes cannot immediately be taken as a reflection of the quality of care that patients receive in these centres: it may be that the case mix at these centres differs from other centres in ways not captured by ANZDATA and therefore the mortality prediction model, or that the observed apparent unusual performance is due to poor quality of data reporting from the centre to the registry. For example, characteristics such as socio-economic status, functional independence, and depression are not recorded or available for modelling - the discrimination of our model would likely be improved through inclusion of additional patient-level characteristics that are out of the control of facilities. The only available marker of kidney function collected by ANZDATA at dialysis start is serum creatinine, which was included in the model for mortality. Other biochemical markers, such as calcium and phosphate, are only recorded at the end of each calendar year, and as they may be modifiable by dialysis centres cannot be included in the risk adjustment model. These points highlight the difficulties in assessment of dialysis centre performance using administrative databases. However, in the absence of a “perfect” data collection incorporating every possible potential confounder, evaluation of outcomes of clinical care needs to be performed on available data.

The likelihood of residual confounding means that log-SMRs here will not definitively identify “good” or “poor” performing units. Rather they identify facilities where further appraisals of processes of care might be considered including more in-depth data analysis. In addition to further investigation of the characteristics of centres with poor performance, the characteristics of centres with unusually good levels of performance should also be investigated. Centre characteristics such as staffing levels, years of operation, and the population they serve should be considered. It is possible that such centres have exemplary processes of care that could be implemented in other centres.

In the end-stage kidney disease setting, how transplantation is dealt with can be of critical importance when modelling outcomes. As pointed out in [22], when survival probabilities are of interest, it is appropriate to treat transplantation as a competing risk rather than censoring patients at the time of transplantation. Different dialysis centres have different patient case mixes and practices, and will thus have different rates of transplantation. Hence, it is necessary to account for transplantation appropriately when comparing mortality across centres. The weighting approach that we have used is tantamount to considering a pseudo population in which no patients receive transplantation and instead remain on dialysis. In this way centres with higher levels of transplantation are not unfairly discriminated against due to censoring of the healthiest patients within that centre. Interestingly, and somewhat surprisingly, the application of this weighting approach did not markedly change results. This aligns with previous work on SMRs and transplantation: in [23] it was shown that the correlation between standardised transplantation ratios and SMRs was very low, although it must be noted that informative censoring due to transplantation was not accounted for in the calculation of SMRs in that work.

Currently, ANZDATA provides annual performance reports to its contributing hospitals, reporting SMRs calculated using Poisson random effect regression models: examples of these reports are available online (http://www.anzdata.org.au/v1/dialysis_hospitalreport.html). Our results are in contrast to reports from ANZDATA comparing the performance of centres from 2008 to 2013, which did not identify any centres as having unusual performance. Some authors have previously observed that different SMR methodologies tend to give similar SMRs [24], but this does not seem to hold for our approach and the ANZDATA approach. Although there are differences between the patients included in ANZDATA centre report and our analyses, the differences between the results of our analysis and those of ANZDATA are likely to be primarily due to differences in statistical methodology, including the choice of SMR considered. The approach we took, fitting a pooled random effects logistic regression model for mortality, weighting to account for the competing risk of kidney transplantation, has advantages over the Poisson random effect model fitted by ANZDATA; although our approach is admittedly more complex. The weighting approach we take essentially results in estimating SMRs amongst a population in which no-one stops haemodialysis to have a transplant (or to start peritoneal dialysis), and thus allows for a fairer comparison of mortality where the rates and patterns of transplantation differ across centres. Our model deals with time to mortality on a finer scale than the ANZDATA model; splitting survival time into 30 day blocks rather than one-year blocks. Another advantage of our model is that includes spline terms for time since dialysis start, and thus allows for a more flexible underlying risk of mortality than does the model fitted by ANZDATA.

The SMR considered here is an indirectly standardised rate: the actual outcomes of patients within a centre are compared to the hypothetical outcomes for those same patients, had those patients been treated at the “average” centre instead of their own centre. The performance of each centre is thus assessed on the basis of the outcomes of the patients actually treated at that centre. Since country is included in the mortality model, the SMR can be thought of as comparing the observed number of deaths within a centre to the expected number of deaths had those patients been randomly distributed amongst centres within the same country. This interpretation can be extended to other variables included in the model. We emphasize that SMRs index facility outcomes to the average outcome for the population, and not the ideal outcome or an external standard of excellence. There are no specific SMR thresholds for classifying HD facilities, and no evidence that facilities with statistically outlying SMRs actually have poorer performance around processes of care. Indeed, the “frequentist” approach used here gives little information beyond a signal that outcomes are likely to differ from average. An alternate approach which may address this is the utilisation of a Bayesian approach, although this has not been implemented in the dialysis performance setting at present [25, 26].

The lack of association of unit performance with adherence to guidelines is intriguing. If the intent of guidelines is to facilitate practices that improve mortality outcomes (as is the case with those examined here), then one would expect centres with greater adherence to target guidelines to have improved performance. Explanations for this include insufficient statistical power; the complex (and non-linear) relationship between serum phosphate levels and outcomes; the limitations of the data collection around central venous catheters (the Registry does not collect indication nor duration of their use). The lack of an association is in contrast to previous work showing an association between the number of facility-level goals achieved and mortality outcomes [27].

## Conclusions

In this study, we demonstrate that SMRs are useful to identify facilities that have statistically outlying mortality risk, and identified two such units. However, we emphasize that SMRs serve only as a screening tool, which can be used to trigger further examination of the facilities with measurably anomalous outcomes. The observed variation in mortality rates across Australian and New Zealand HD facilities could not here be attributed to measured patient factors. Moreover, it cannot be attributed to compliance with bi-national clinical practice guidelines, insofar as levels of compliance are not associated with different outcomes.

## Additional file

Additional file 1: Contains: Additional Item 1: SMR formula. **Figures S1-3.** boxplots of patient ages, serum creatinine levels, and BMI by dialysis centre. **Figures S4–8.** proportions of patients in each centre with comorbidities, diabetes type I or II, referred late to a nephrologist, in each race category, with each primary renal disease category at dialysis start. **Figure S9.** proportion of patients commencing dialysis in each year 2008–2010 by dialysis centre. **Figures S10–12.** funnel plots for log-SMRs from sensitivity analyses. **Figure S13.** proportion of measurements in each centre consistent with KHA-CARI guidelines, plotted against log-SMR. **Figure S14.** proportion of patients with arteriovenous fistulas in each centre, plotted against log-SMR (PDF 1045 kb)

## Abbreviations

ANZDATA Registry: Australia and New Zealand Dialysis and Transplant Registry
BMI: Body mass index
KHA-CARI Guidelines: Kidney Health Australia – Caring for Australasians with Renal Impairment Guidelines
PD: Peritoneal dialysis
SMR: Standardised mortality ratio
