# Supplementary material for: Comparing dialysis centre mortality outcomes across Australia and New Zealand: identifying unusually performing centres 2008–2013
Source: BMC Health Serv Res. 2018 Dec 29;18:1007. doi: 10.1186/s12913-018-3832-0 (PMC6311072; doi:10.1186/s12913-018-3832-0)
Supplement: Supplementary file 1 — Contains: Additional Item 1: SMR formula. Figures S1-3. boxplots of patient ages, serum creatinine levels, and BMI by dialysis centre. Figures S4–8. proportions of patients in each centre with comorbidities, diabetes type I or II, referred late to a nephrologist, in each race category, with each primary renal disease category at dialysis start. Figure S9. proportion of patients commencing dialysis in each year 2008–2010 by dialysis centre. Figures S10–12. funnel plots for log-SMRs from sensitivity analyses. Figure S13. proportion of measurements in each centre consistent with KHA-CARI guidelines, plotted against log-SMR. Figure S14. proportion of patients with arteriovenous fistulas in each centre, plotted against log-SMR (PDF 1045 kb) [file 12913_2018_3832_MOESM1_ESM.pdf]

Additional File

To accompany:

Comparing dialysis centre mortality outcomes across Australia and New Zealand: identifying unusually performing centres 2008-2013

Jessica Kasza, Kevan R Polkinghorne, Rory Wolfe, Stephen P McDonald, Mark R Marshall

Additional Item 1. SMR formula

For centre $i = 1, \dots, K$ with patients $j = 1, \dots, n_i$ experiencing mortality outcomes o_{ij} (0=alive, 1=dead), the SMR is calculated as

$$SMR_i = \frac{\sum_{j=1}^{n_i} o_{ij}}{\sum_{j=1}^{n_i} \sum_{k=1}^{K} P(\text{mortality of patient } j \text{ if treated in centre } k)/K}$$

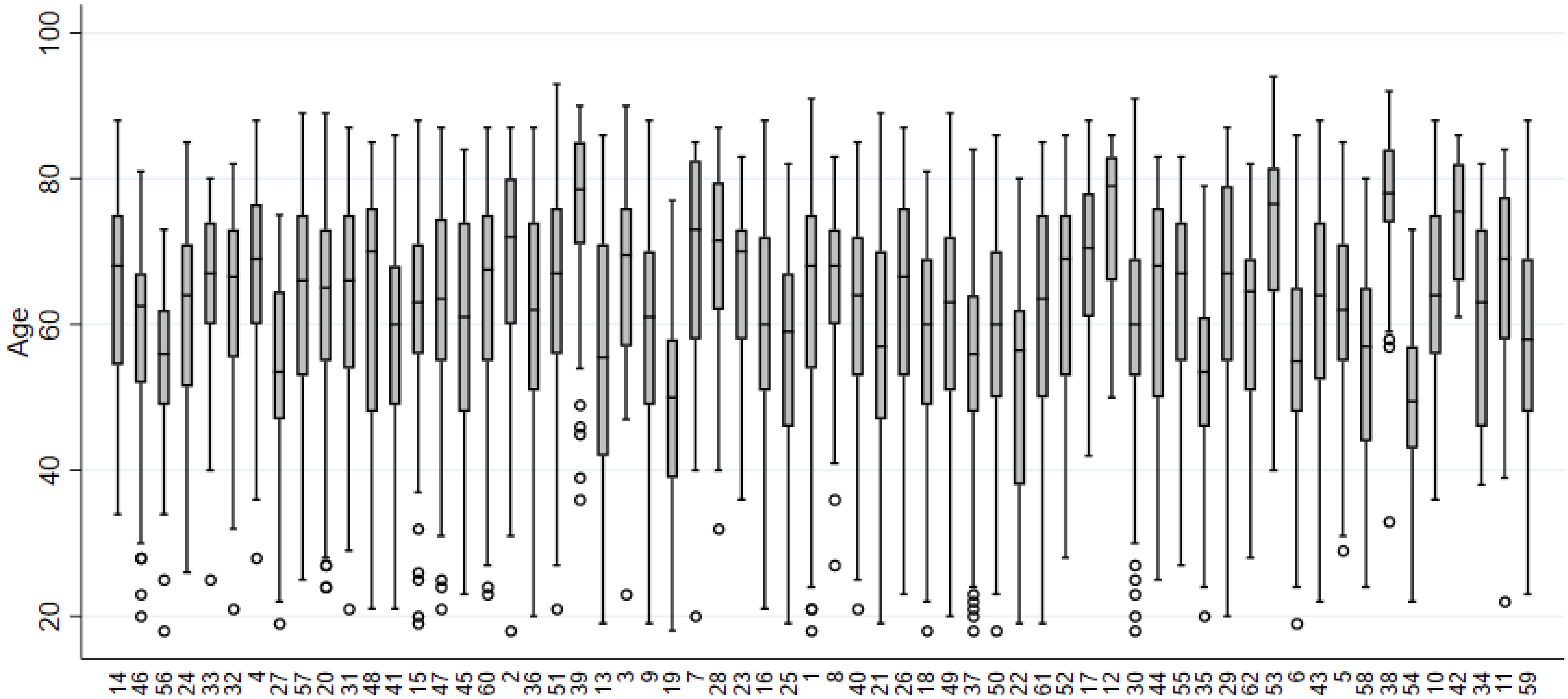

Additional Figure 1: Boxplots of patient ages within each centre. Centres are ordered by log-SMR (smallest to largest), and labelled with random identifying numbers.

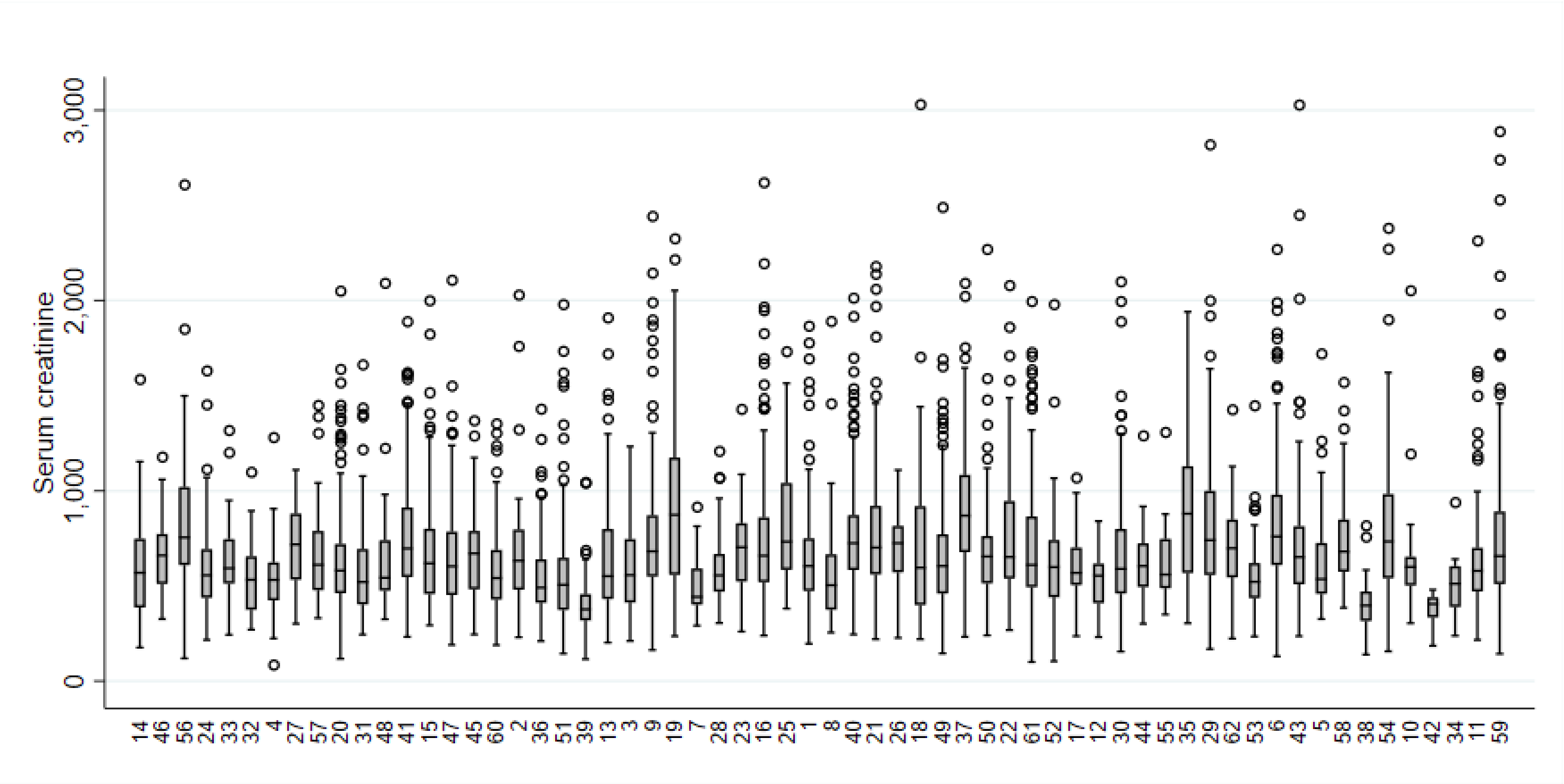

Additional Figure 2: Boxplots of patient serum creatinine levels within each centre. Centres are ordered by log-SMR (smallest to largest), and labelled with random identifying numbers.

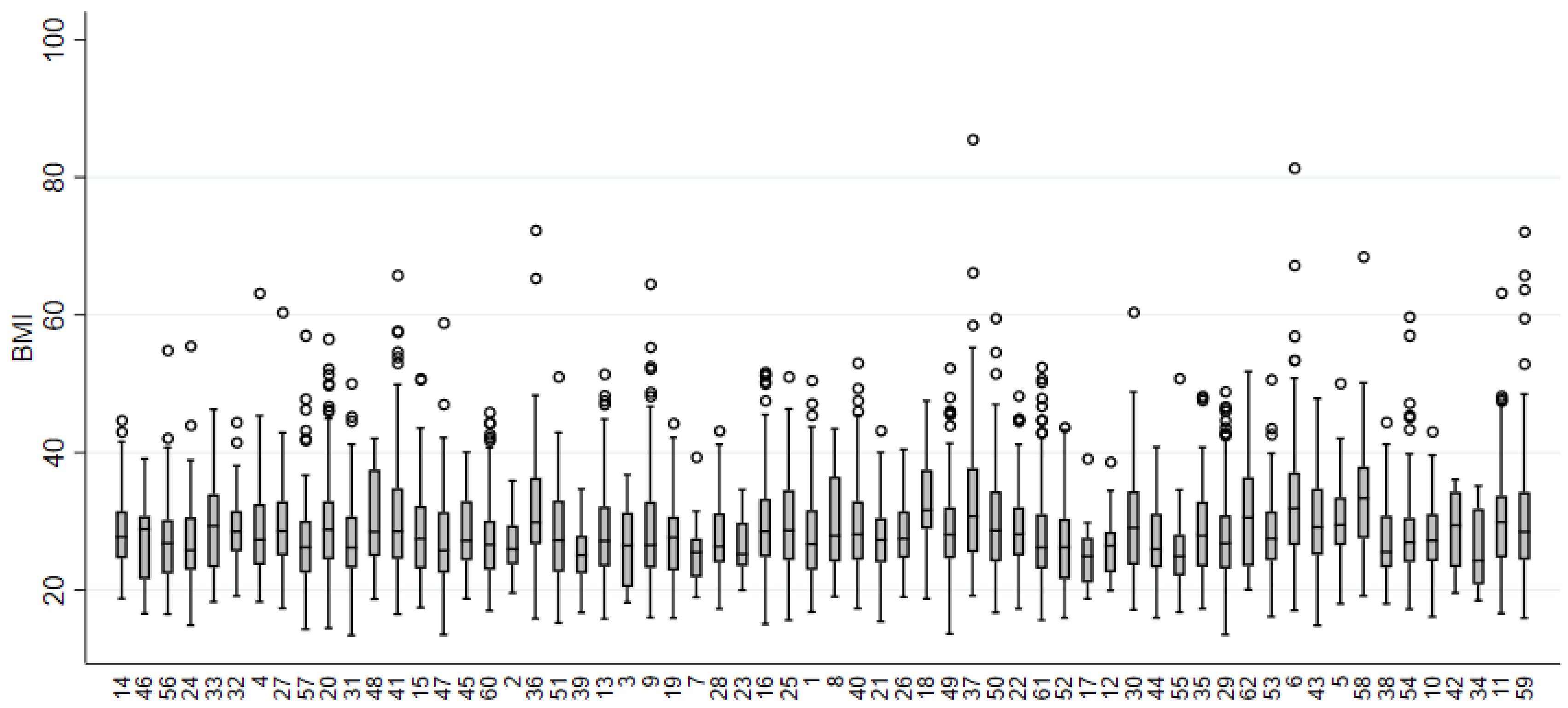

Additional Figure 3: Boxplots of patient BMIs within each centre. Centres are ordered by log-SMR (smallest to largest), and labelled with random identifying numbers.

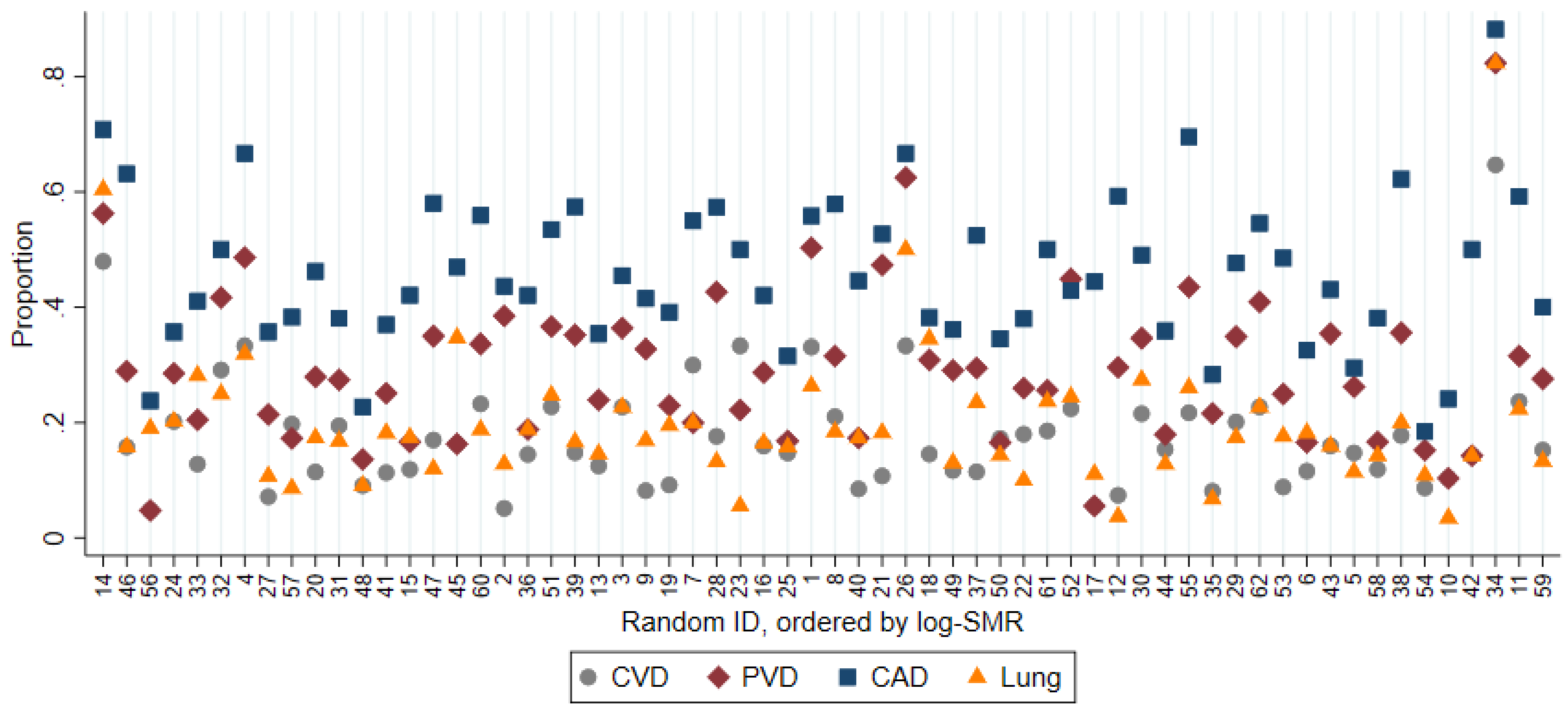

Additional Figure 4: Proportion of patients with comorbidities, within each dialysis centre. Centres are ordered by log-SMR (smallest to largest) labelled with random identifying numbers. CVD: cerebrovascular disease; PVD: peripheral vascular disease; CAD: coronary artery disease; Lung: lung disease.

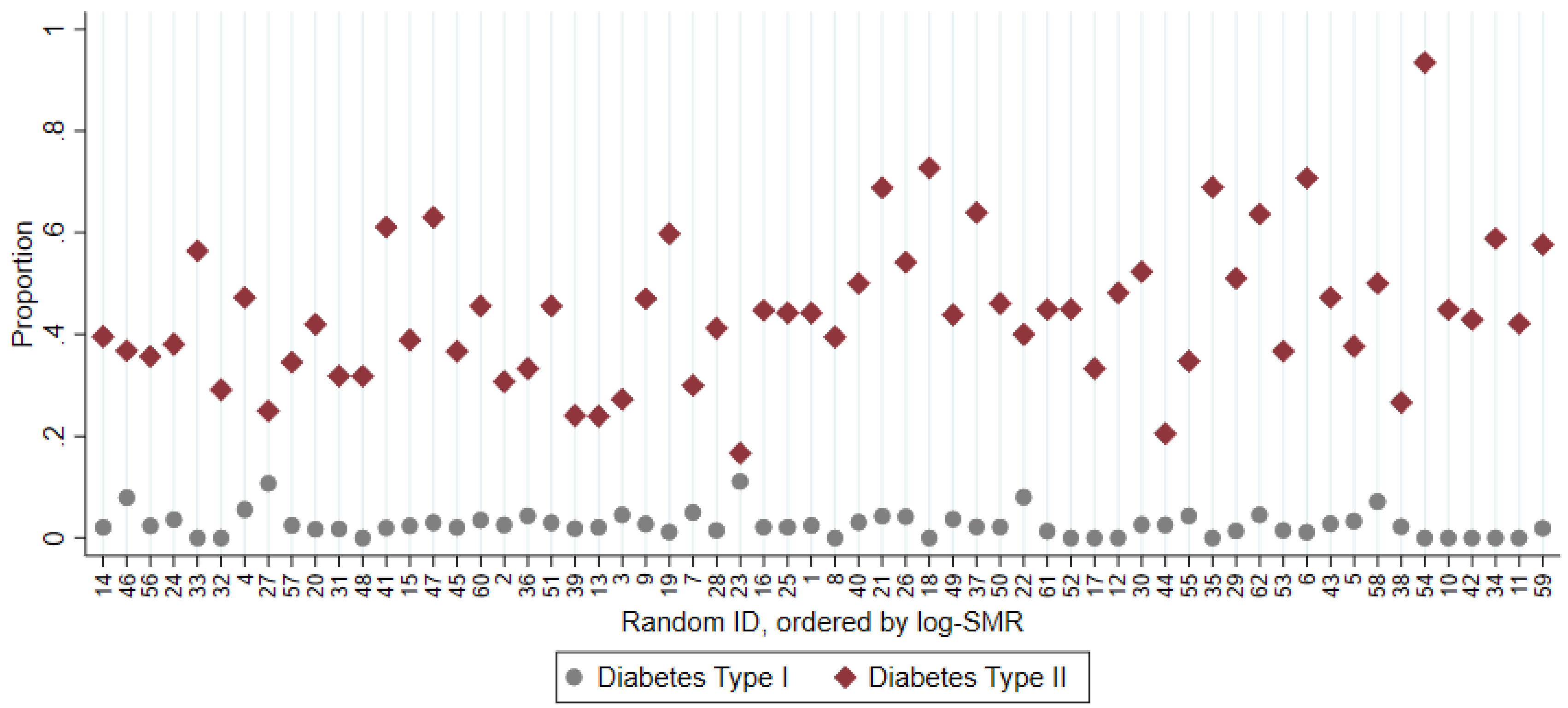

Additional Figure 5: Proportion of patients with diabetes type I or II, within each dialysis centre. Centres are ordered by log-SMR (smallest to largest), and are labelled with random identifying numbers.

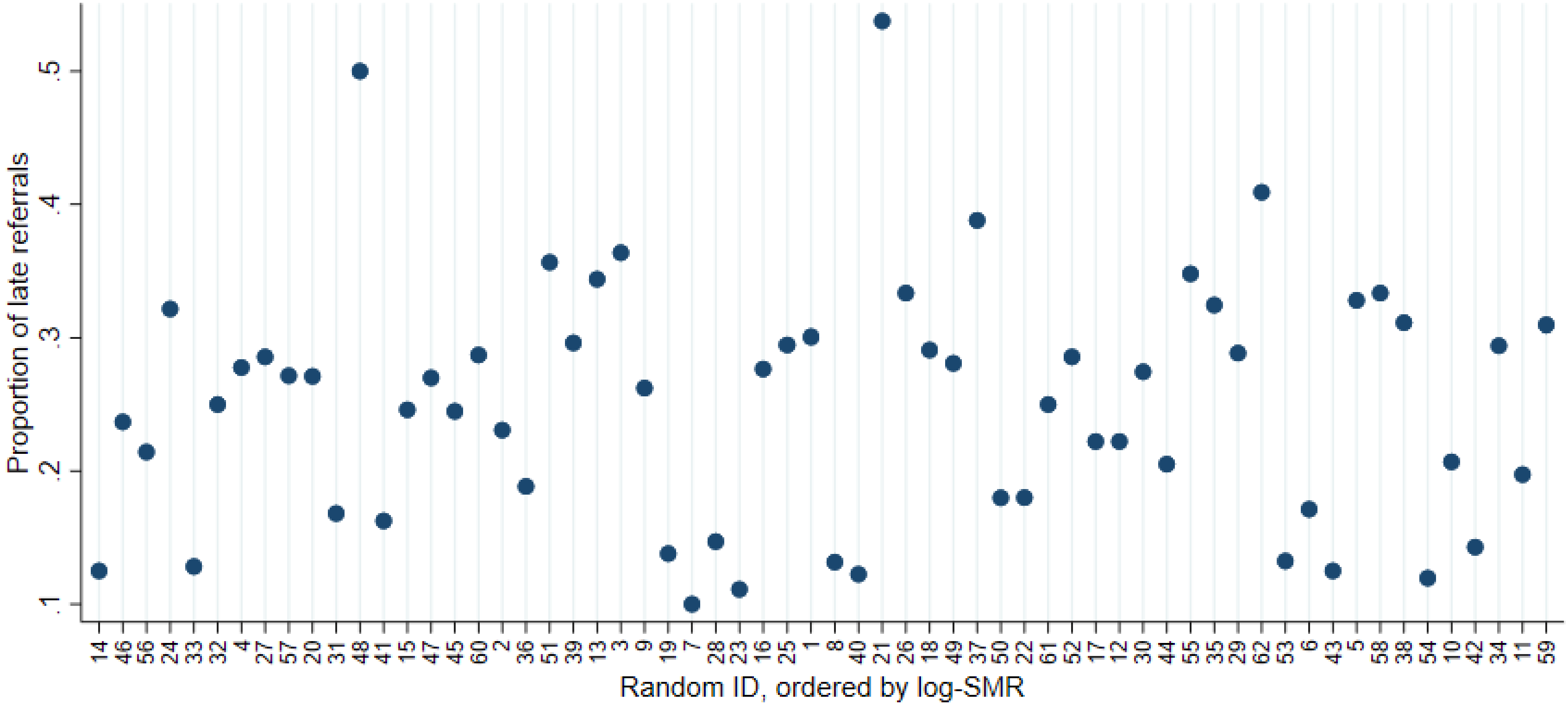

Additional Figure 6: Proportion of patients referred late to a nephrologist (within 3 months of dialysis start), within each dialysis centre. Centres are ordered by log-SMR (smallest to largest) and are labelled with random identifying numbers.

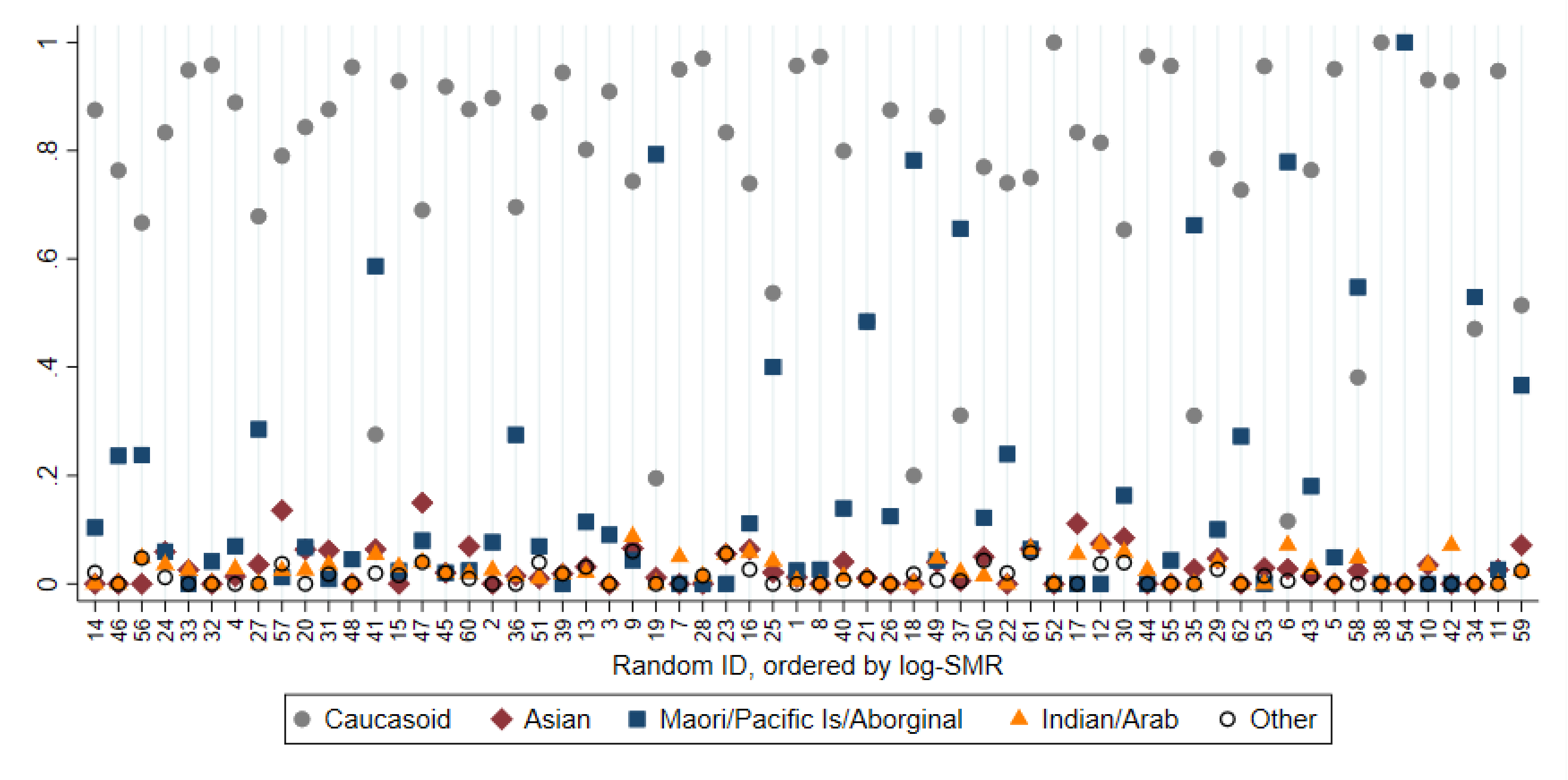

Additional Figure 7: Proportion of patients in each race category, within each dialysis centre. Centres are ordered by log-SMR (smallest to largest), and are labelled with random identifying numbers.

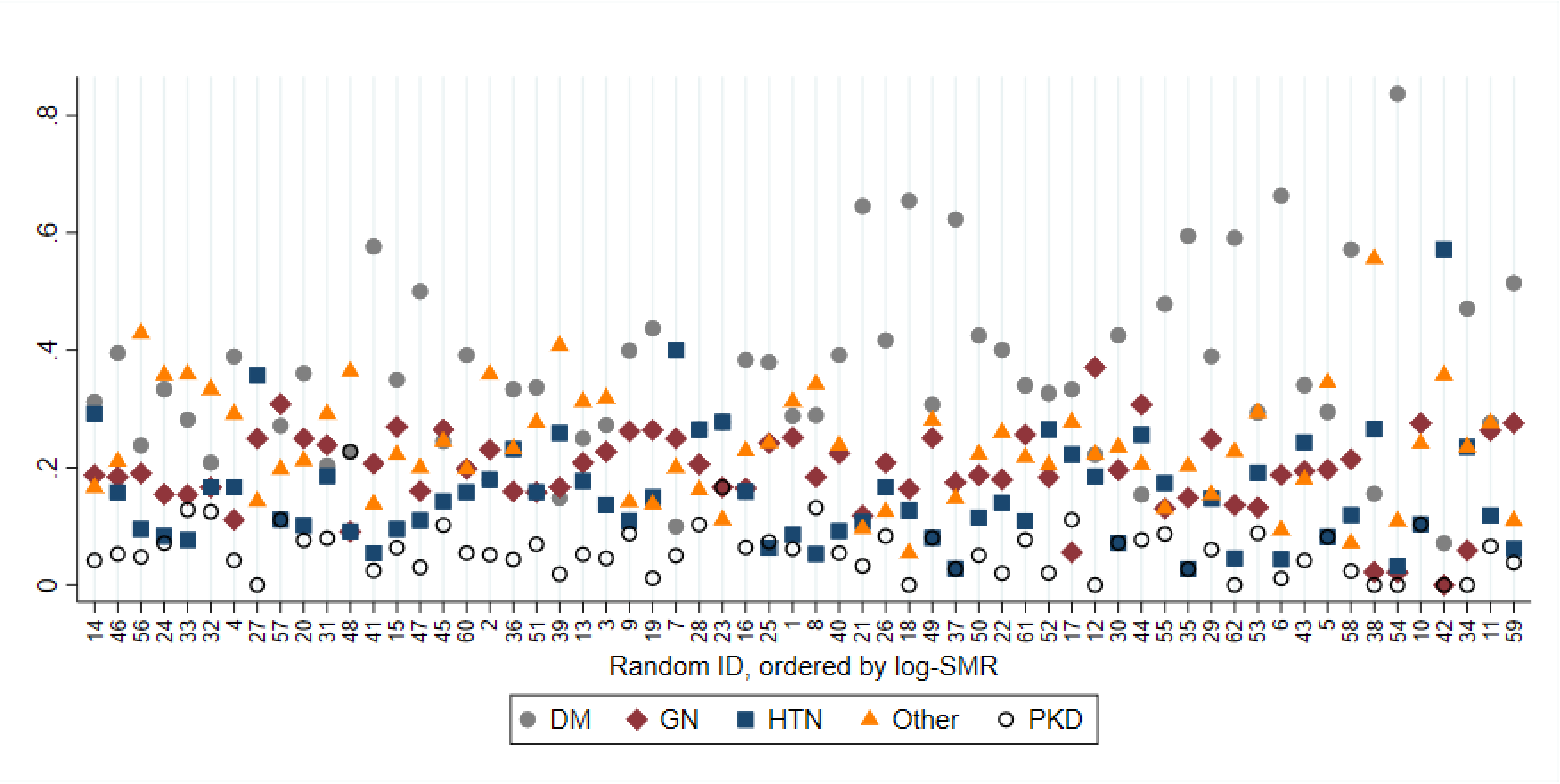

Additional Figure 8: Proportion of patients with each primary renal disease category, within each dialysis centre. Centres are ordered by log-SMR (smallest to largest), and are labelled with random identifying numbers. DM: diabetes mellitus; GN: glomerulonephritis; HTN: hypertensive nephropathy; PKD: polycystic kidney disease.

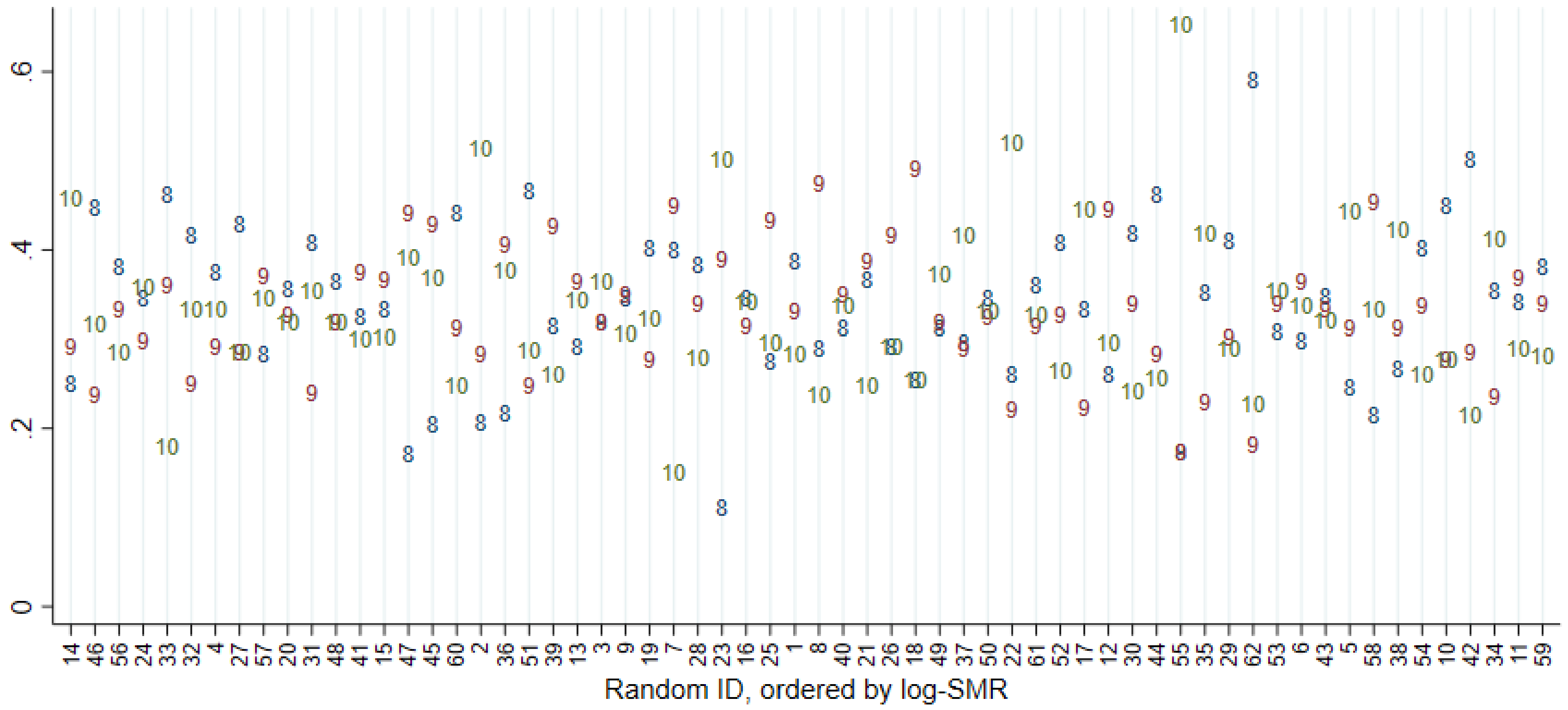

Additional Figure 9: Proportion of patients starting dialysis in each year 2008-2010 (2008=8, 2009=9, 2010=10), within each dialysis centre. Centres are ordered by log-SMR (smallest to largest), and are labelled with random identifying numbers.

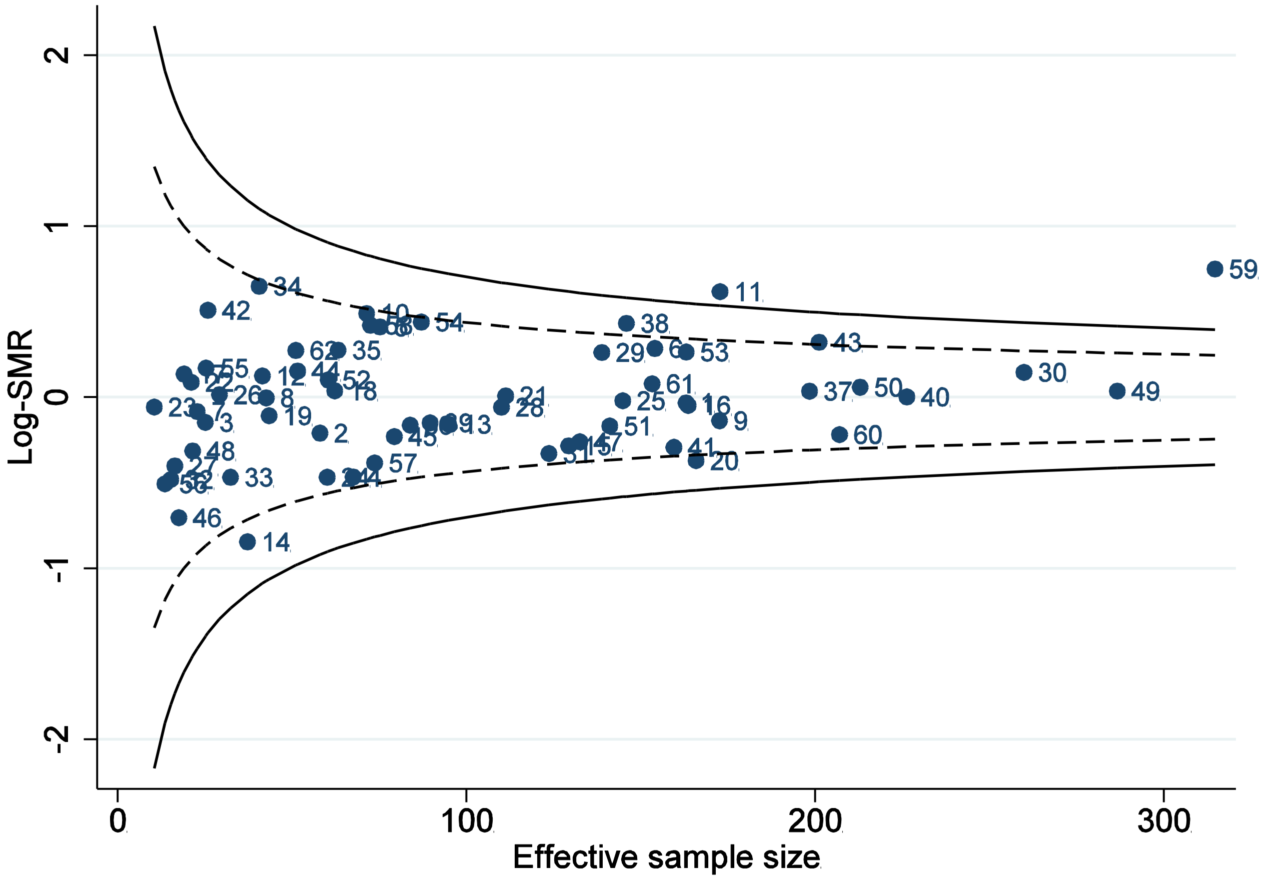

Additional Figure 10: Funnel plot for log-SMRs obtained using the random effects pooled logistic model for mortality, without accounting for the informative censoring events of kidney transplantation and switch to peritoneal dialysis. The dashed lines are the classical 95% prediction limits when multiple comparisons are not adjusted for; the solid lines control the false discovery rate at 5%. Centres are labelled with random identifying numbers.

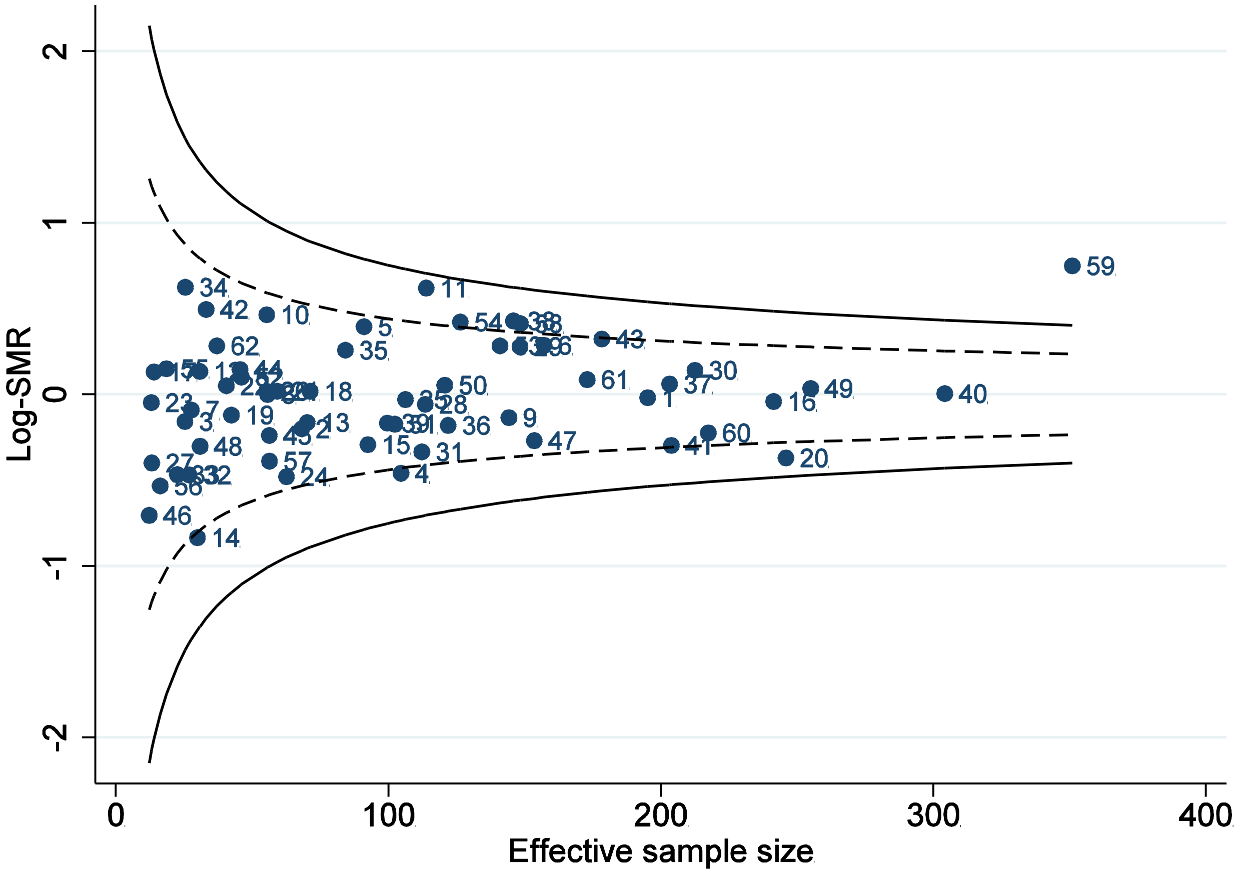

Additional Figure 11: Funnel plot for log-SMRs obtained using the random effects pooled logistic model for mortality, accounting for censoring at the time of kidney transplant and switch to peritoneal dialysis using the weighting approach, excluding patients who commenced peritoneal dialysis within 90 days of dialysis start. The dashed lines are the classical 95% prediction limits when multiple comparisons are not adjusted for; the solid lines control the false discovery rate at 5%. Centres are labelled with random identifying numbers.

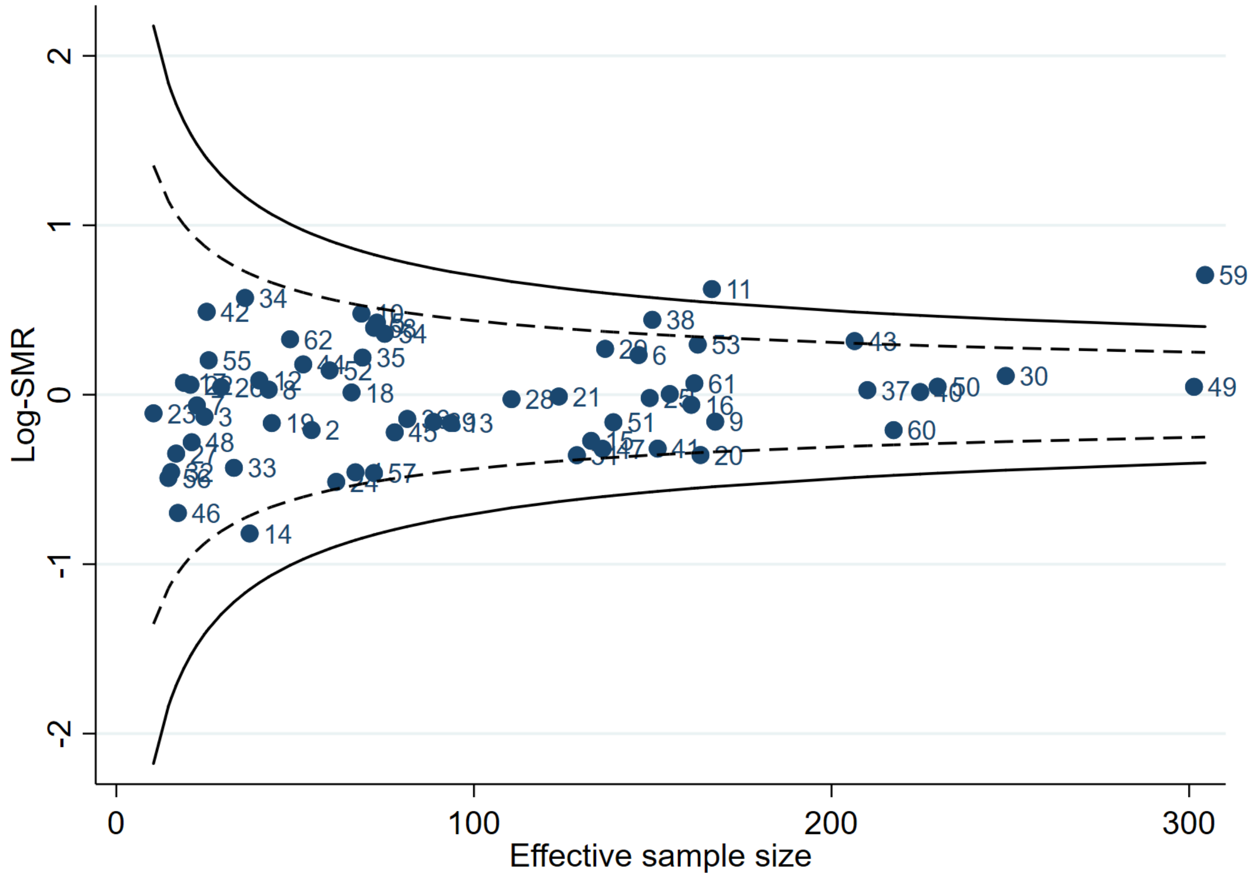

Additional Figure 12: Funnel plot for log-SMRs obtained using the random effects pooled logistic model for mortality, accounting for censoring at the time of kidney transplant and switch to peritoneal dialysis using the weighting approach, excluding race from the risk adjustment models. The dashed lines are the classical 95% prediction limits when multiple comparisons are not adjusted for; the solid lines control the false discovery rate at 5%. Centres are labelled with random identifying numbers.

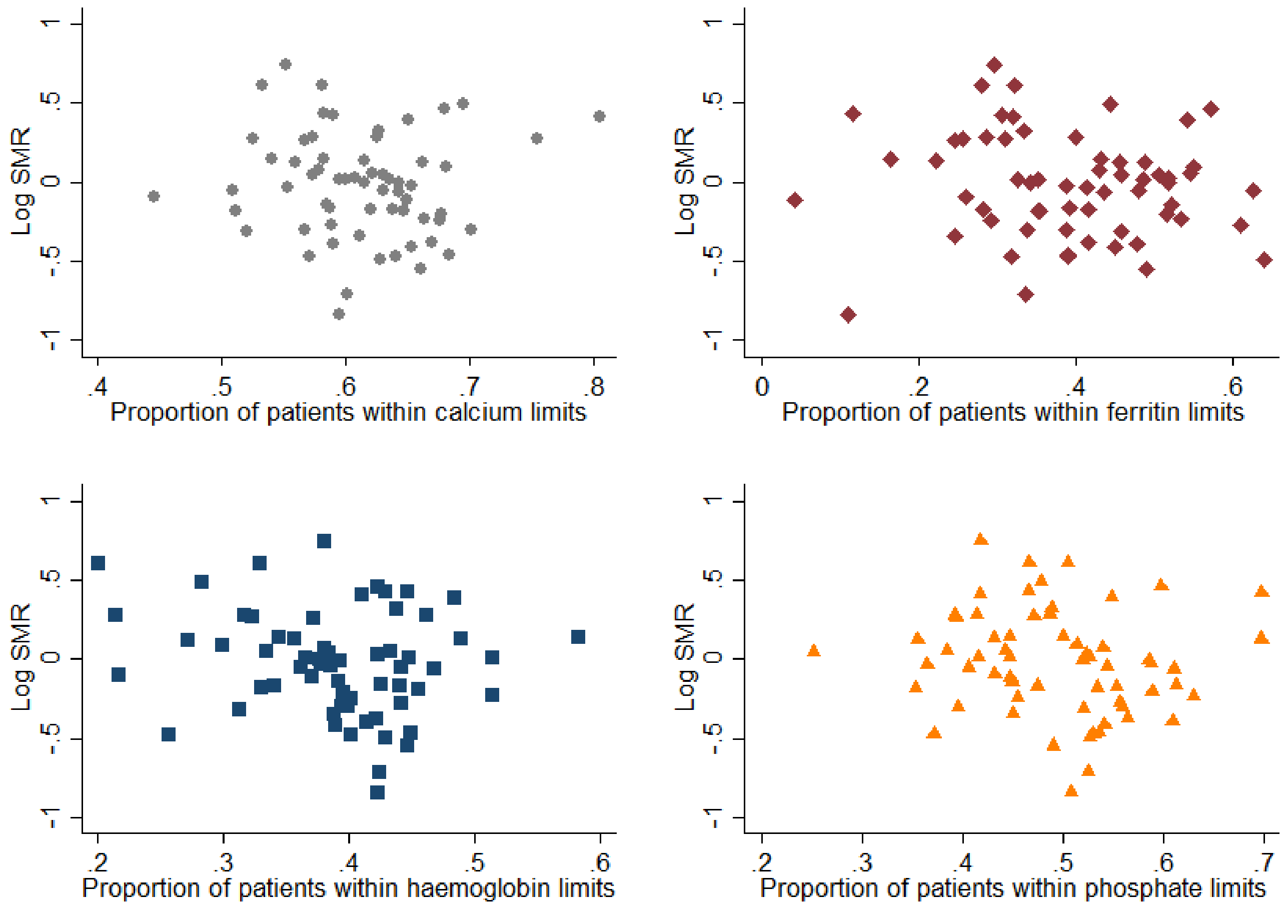

Additional Figure 13: Scatter plots of the proportion of measurements consistent with KHA-CARI guidelines for calcium, ferritin, haemoglobin and phosphate concentrations, within each dialysis centre, against log-SMR.

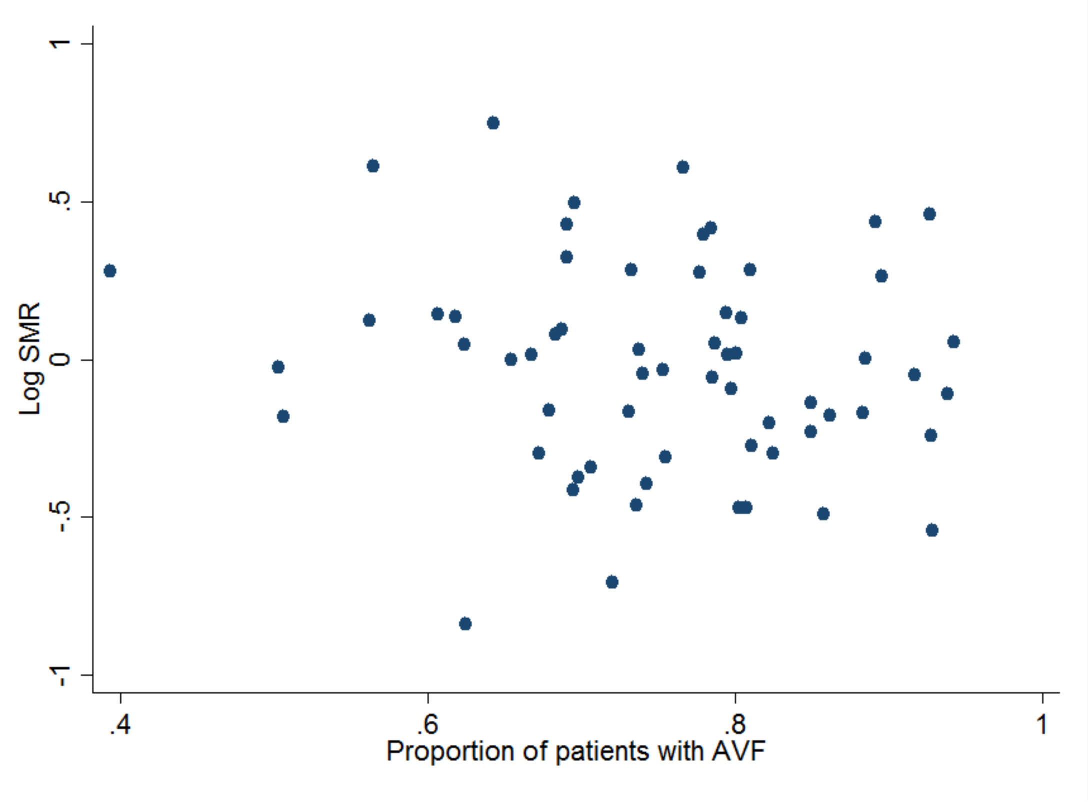

Additional Figure 14: The proportion of patients with arteriovenous fistulas within each dialysis centre against log-SMR.
